# Antimicrobial stewardship in Scotland: impact of a national programme

**DOI:** 10.1186/2047-2994-1-7

**Published:** 2012-02-03

**Authors:** Dilip Nathwani, Jacqueline Sneddon, Andrea Patton, William Malcolm

**Affiliations:** 1 Ninewells Hospital & Medical School, Dundee, DD19SY, UK; 2 Scottish Medicines Consortium, Delta House, 50 West Nile Street, Glasgow G1 2NP, UK; 3 Healthcare Improvement Scotland, Elliott House, 8-10 Hillside Crescent, Edinburgh EH7 5EA, UK; 4 NHS National Services Scotland, Health Protection Scotland, Meridian Court, 5 Cadogan Street, Glasgow G2 6QE, UK

**Keywords:** Antimicrobial stewardship, *Clostridium difficile *infection, prescribing indicators, quality improvement

## Abstract

**Backgound:**

The Scottish Antimicrobial Prescribing Group (SAPG) was established by the Scottish Government in 2008 to lead the first national initiative to actively address antimicrobial stewardship. Healthcare associated infection (HAI) is a priority in Scotland and the work of SAPG contributes to the national HAI Delivery Plan. SAPG's early work has focused on restricting the use of antibiotics associated with a high risk of *Clostridium difficile *infection (CDI) and development of national prescribing indicators to support reduction of CDI.

**Findings:**

Scottish Antimicrobial Prescribing Group has developed prescribing indicators for hospital and primary care, which are measured and reported in all 14 NHS board areas. Improvement in compliance with the indicators has been demonstrated with resultant reductions in CDI rates and no adverse effect on mortality or antimicrobial resistance patterns.

**Conclusions:**

The establishment of a Scottish national antimicrobial stewardship programme has made a significant contribution to the HAI agenda, particularly in relation to CDI. The programme is supported by local antimicrobial teams, a national framework for education, surveillance of antimicrobial use and resistance and sharing of data for improvement. Antimicrobial stewardship has been integrated with other national programmes on patient safety and quality improvement.

## Background

The Scottish Antimicrobial Prescribing Group (SAPG) [1] was formed in March 2008 by the Scottish Government Health Department (SGHD) to implement the recommendations of the Scottish Management of Antimicrobial Resistance Action Plan (ScotMARAP) [2]. SAPG operates as a multidisciplinary national clinical forum and its primary objectives are to co-ordinate and deliver a national framework for antimicrobial stewardship to enhance the quality of antimicrobial prescribing and management in Scotland.

## Materials and methods

SAPG was established to lead the first national initiative to actively address antimicrobial stewardship. Collaboration with key stakeholders at national level and development of local (NHS board level) leadership through Antimicrobial Management Teams has been essential to develop national consensus and implement key stewardship interventions. This has been combined with the development of national and local systems and a framework for collecting and disseminating data on antibiotic prescribing and resistance surveillance and a framework for educational material to support the healthcare professionals in the workplace.

Healthcare associated infection (HAI) is a priority for the Scottish Government and the work of SAPG contributes to the national HAI Delivery Plan. Reduction of *Clostridium difficile *infection (CDI) is a key objective for NHS Scotland and in 2009 a target was set for all NHS boards to reduce their rate by 30% (later increased to 50%) in patients over 65 years. Much of the focus of SAPG's early work has therefore been to reduce the use of antibiotics associated with a high risk of CDI across hospital and primary care settings. SAPG developed national prescribing indicators based on compliance with local antibiotic policies to support reduction of CDI:

### Empirical Prescribing in hospital

Indication for treatment recorded in the patient medical record and antibiotic choice is compliant with the local Antimicrobial Prescribing Policy - target ≥ 95%

### Primary care prescribing

Seasonal variation in quinolone use, consumption of quinolones in winter months is ≤ 5% greater than consumption in summer months.

## Results

National surveillance data confirm that there has been a progressive reduction in CDI throughout Scotland and that all NHS boards have exceeded the original target of 30% reduction. The national rate has fallen from 1.5 to 0.32 cases per 1000 acute occupied bed days [3]. Improvement in the quality of antimicrobial prescribing in hospital and primary care has made a significant contribution to this reduction.

### Changes in antibiotic use

National results for the hospital and primary care CDI prescribing indicators are shown in Additional file 1, Table S1 and Additional File 2, Figure S1

### Impact on outcomes

#### Clostridium difficile infection (CDI)

There is a temporal association between this reduction and the introduction of restrictive antimicrobial policies in line with SAPG guidance. The true impact of this intervention is being evaluated using a time series analysis and results from one region (NHS Tayside) show a reduction in total CDI post-intervention of 54% in Medicine and 50% in Surgery [4]. The attendant cost avoidance due to this reduction in CDI in is estimated at more than £500 K per year (based on £4 K per CDI episode [5]).

#### Unintended consequences

National surveillance of antimicrobial resistance shows no significant increases in resistance to key Gram-negative organisms and reduced resistance to third generation cephalosporins in *E. coli*. Aminoglycoside-related toxicity is also being prospectively evaluated.

#### Mortality

Crude 30-day all-cause mortality (from admission) was analysed for both NHS Tayside cohorts. No change was observed following the introduction of the restricted antibiotic policy. Subgroup analysis by age and Charlson Co-morbidity Index score showed no changes in mortality following the introduction of the policy. Further analysis using adjusted 30-day mortality in patients who have had blood cultures taken as an outcome indicator for sepsis management and antibiotic stewardship interventions is also being undertaken.

## Conclusion

The establishment of a Scottish National Antimicrobial Stewardship Programme has made a significant contribution to the HAI agenda, particularly in relation to CDI [6]. Providing local clinical prescribing leadership through Antimicrobial Management Teams and organisational accountability through agreed national targets for prescribing have been key success factors in bringing about this change. This is supported by a national framework for education, timely sharing of data using the Institute for Healthcare Improvement Extranet and a range of local improvement strategies in collaboration with local infection prevention and quality improvement teams. Aligning antimicrobial stewardship with patient safety and quality improvement which are key elements of the national Quality Strategy [7] has also been particularly helpful. During the next three years we aim to consolidate our success and embark upon further collaborative improvement work in primary care prescribing, surgical prophylaxis and management of sepsis, the latter being a significant cause of hospital mortality and morbidity and a national priority for 2012.

## Competing interests

The authors declare that they have no competing interests.

## Authors' contributions

All authors are members of the Scottish Antimicrobial Prescribing Group (SAPG). DN has been chair of SAPG since 2008 and JS has been Project Lead for SAPG since 2008. DN and JS have made substantial contributions to the conception and design of the SAPG work programme including development of the prescribing indicators to support reduction of CDI. AP is responsible for developing and maintaining the data management system for measuring and reporting the hospital prescribing indicators and for performing the analysis of data from NHS Tayside to confirm the impact of changes in antimicrobial use on CDI and mortality. WM made a significant contribution to development of the primary care prescribing indicator and is responsible for evaluation and reporting of the data for this indicator. All authors have read and approved the final manuscript.

## Authors' information

DN is Chair of SAPG and is a Consultant Physician in Infectious Diseases and Honorary Professor of Infection, Ninewells Hospital and Medical School, Dundee, UK

JS is Project Lead for SAPG and has a hospital pharmacy background with a PhD in medicinal chemistry and an MSc in Clinical Pharmacy.

AP is an Information Analyst with an MSc in Applied Statistics.

WM is Pharmaceutical Adviser at Health Protection Scotland and has a background in pharmaceutical public health and primary care pharmacy.

## Supplementary Material

Additional file 1 **Table S1 Compliance with empirical prescribing in Medical Admission Units April - June 2011**. Summary of data for national prescribing indicator for empirical prescribing in hospital. Click here for file

Additional file 2 **Figure S1 Primary care seasonal variation of quinolone use by NHS board 2008-2011**. Annual data for national prescribing indicator for primary care. Click here for file
