# Supplementary material for: Antimicrobial stewardship in Scotland: impact of a national programme
Source: Antimicrob Resist Infect Control. 2012 Feb 3;1:7. doi: 10.1186/2047-2994-1-7 (PMC3436612; doi:10.1186/2047-2994-1-7)
Supplement: Additional file 1 — Table S1 Compliance with empirical prescribing in Medical Admission Units April - June 2011. Summary of data for national prescribing indicator for empirical prescribing in hospital. [file 2047-2994-1-7-S1.DOC]

**Additional file 1**

Title: Table S1 Compliance with empirical prescribing in Medical Admission Units April – June 2011

Description: Summary of data for national prescribing indicator for empirical prescribing in hospital

| **Indication Documented** | | | **Policy Compliant** | | | |
| --- | --- | --- | --- | --- | --- | --- |
| **Sample Size** | **Median % Compliance** | **Boards Achieving Target (>=95%)** | **Sample Size** | **Median % Compliance** | **Boards Achieving Target (>=95%)** |  |
| **1368** | **100%** | **10 of 14** | **1174** | **90%** | **4 of 14** |  |
