# Supplementary material for: Antimicrobial stewardship in Scotland: impact of a national programme
Source: Antimicrob Resist Infect Control. 2012 Feb 3;1:7. doi: 10.1186/2047-2994-1-7 (PMC3436612; doi:10.1186/2047-2994-1-7)

**Additional file 2**

Title: Figure S1 Primary care seasonal variation of quinolone use by NHS board 2008-2011

Description: Annual data for national prescribing indicator for primary care


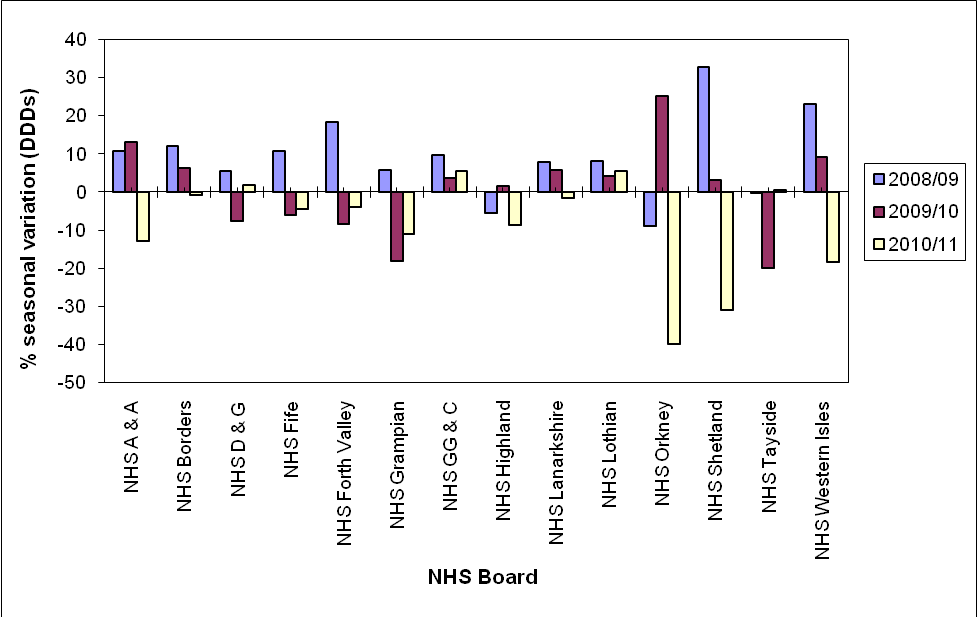

Supplement: Additional file 2 — Figure S1 Primary care seasonal variation of quinolone use by NHS board 2008-2011. Annual data for national prescribing indicator for primary care. [file 2047-2994-1-7-S2.DOC]
